# Perceived overqualification and deviant innovation behavior: The roles of creative self-efficacy and perceived organizational support

**DOI:** 10.3389/fpsyg.2023.967052

**Published:** 2023-01-27

**Authors:** Lu Lu, Tanghong Luo, Yajun Zhang

**Affiliations:** School of Business Administration, Guizhou University of Finance and Economics, Guiyang, China

**Keywords:** perceived overqualification, creative self-efficacy, perceived organizational support, deviant innovation behavior, moderated mediation

## Abstract

Employees’ perceived overqualification (POQ) is prevalent in organizations and has drawn increasing attention from both researchers and practitioners. Drawing from social cognitive theory, the purpose of this study is to extend existing understanding of the consequences of POQ by examining how and when POQ leads to deviant innovation behavior. This study hypothesizes that employees’ POQ indirectly impacts deviant innovation through enhanced creative self-efficacy (CSE), and that perceived organizational support (POS) strengthens this indirect relationship. Using data collected from 286 employees in China at two time points, this study found support for our hypotheses that POQ is positively related to CSE, and that CSE mediates the relationship between POQ and deviant innovation behavior. In addition, this study found that POS moderates the relationship between POQ and CSE, as well as the indirect effect of POQ on deviant innovation behavior *via* CSE. The theoretical and practical implications of our findings and future research directions are discussed.

## 1. Introduction

As the worldwide economy contracts, the realization by employees that finding a job that matches their qualifications has become more difficult and the feeling by employees that they hold more knowledge, skills, and abilities than their position requires has become a universal phenomenon in organizations. Both researchers and practitioners have increasing interest in perceived overqualification (POQ), which is defined as the perception by employees that their qualifications, such as cognitive abilities, job-related skills, and formal education, exceed the requirements of the job (Erdogan and Bauer, 2009). These surplus qualifications enable employees to perceive mismatch between skills and the job, and thus they are more likely to experience reduced job satisfaction and affective commitment (Maynard et al., 2006; Arvan et al., 2019), in addition to increased relative deprivation (Erdogan et al., 2018). In response to this perceived mismatch, overqualified employees are likely to have a high turnover rate and higher likelihood of resignation (Erdogan and Bauer, 2009; Wang, 2018). Overqualified employees might also engage in counterproductive work behaviors (Luksyte et al., 2011; Liu et al., 2015; Schreurs et al., 2021) and cyberloafing behavior (Cheng et al., 2020; Andel et al., 2022).

Despite these negative consequences of employees’ POQ, recent research indicated that POQ can also result in some positive outcomes. For example, overqualified employees may have positive self-evaluation (Ma et al., 2020), and thus are more likely to exhibit proactive behaviors (Zhang et al., 2016). Overqualified employees can also be creative under some circumstances (Luksyte and Spitzmueller, 2016), experience more subjective vitality through job crafting (Zhang F. et al., 2021), and enhance their task performance through task mastery (Lee et al., 2021). These contradictory findings on the consequences of POQ warrant further exploration of whether the positive evaluation and sense of superiority associated with POQ would enable employees to take risks to show their capacity to contribute while breaking some organizational rules.

The objective of this study was to examine the influence of POQ on deviant innovation behavior, as well as its underlying mechanism and boundary condition. Deviant innovation behavior is defined as employees’ violation of managerial order or supervisory control to achieve innovation, which consists of both constructive creativity and destructive deviance (Mainemelis, 2010). Constructive creativity suggests it is a creative process in which employees have new ideas and then promote the implementation of ideas (Xu and Zhao, 2020), and destructive deviance means employees cannot achieve their own innovation goals through formal channels and may turn to informal ways (Zhang and Tu, 2022). Overqualified employees may have higher aspirations for advancement and expanded job tasks that they feel they deserve (Erdogan and Bauer, 2009), and thus they often feel dissatisfaction with mismatch between job demands and skills (Johnson et al., 2002; Maynard et al., 2006; Fine and Nevo, 2008). To mitigate this dissatisfaction, such employees may take actions to change their employment to a position that could better utilize their qualifications (Harari et al., 2017). Another way for employees to address this mismatch is to continue to pursue ideas that were rejected by organizations until they achieve success (Lin et al., 2017).

Here the current study further explores the mechanism underlying the impact of POQ on deviant innovation behavior based on social cognitive theory (Bandura, 1986). One of the central ideas of social cognitive theory is that human behavior is influenced by “people’s judgments of their capabilities to organize and execute courses of action required to attain designated types of performances” (Bandura, 1986, p. 391), and such judgment is termed as self-efficacy (Bandura, 1997). Individuals’ self-efficacy could influence whether they will initiate certain behavior, the amount of effort they decide to exert, and the length of their persistence in the face of obstacles (Bandura, 1977). Creative self-efficacy (CSE) is one type of self-efficacy which is defined as the confidence that one has in one’s ability to produce creative outcomes, and plays as a core mechanism for creativity and innovation (Tierney and Farmer, 2002, 2004). Employees with POQ have been shown to typically have positive self-perception regarding their competences and skills in creative domains (Luksyte and Spitzmueller, 2016). In other words, overqualified employees may be more confident about their creative abilities while undertaking deviant innovation behavior. Thus, this study proposes that employees who perceive themselves as overqualified participate in deviant innovation behavior through heightened CSE.

This study also aims to explore the boundary condition under which the influence of POQ would be exacerbated by investigating the moderating role of perceived organizational support (POS), which reflects employees’ general beliefs about the extent to which the organization values their contributions and cares about their wellbeing (Eisenberger et al., 1986). Existing research suggests that the perception of overqualification can be amplified in a supportive work environment, wherein overqualified employees actively redesign their work assignments (Lin et al., 2017). Overqualified employees who have high POS would consider themselves to be valued and their organizational environment to be supportive. Accordingly, they tend to be more confident in their creative abilities and are more likely to take risks to demonstrate their abilities. Thus, POS would act as a boundary condition for the impact of POQ on CSE.

Taken together, this study aims to answer how and when POQ would lead to deviant innovation behavior by examining the mediating role of CSE in the relationship between POQ and CSE, and the moderating role of POS on the relationship between POQ and CSE.

## 2. Theoretical background and hypotheses development

### 2.1. POQ and CSE

Perceived overqualification refers to the extent to which employees perceive themselves to possess more qualifications, such as education, experience, or skills, than the job requires (Johnson and Johnson, 1997; Johnson et al., 2002). POQ differs from objective overqualification, which usually reflects the mismatch between the qualification of the employee and stated job description, whereas POQ has greater focus on the subjective feelings of employees. Thus, two employees who have the same level of education, experience, or skills and hold the same position may have different POQ. The current study focuses on POQ rather than objective overqualification, because POQ is considered as being more representative of the complexities involved in experiencing overqualification and thus more likely to provide a meaningful prediction of job attitudes and behaviors (Erdogan et al., 2011; Lee et al., 2021).

Based on the general definition of self-efficacy, Tierney and Farmer (2002) proposed the concept of CSE, which they defined as the belief that one has the competence to achieve creative outcomes. Similar to other types of self-efficacy, CSE suggests a psychological state through which a positive judgment of one’s creative abilities is made. As overqualified employees not only possess surplus job skills, but also tend to evaluate their job competence positively (Zhang et al., 2016; Deng et al., 2018), and come to perceive that their qualifications exceed job requirements, they may have more confidence that they can be creative in their work roles. Thus, this study proposes that the POQ of employees could positively influence their CSE for the following reasons.

First, as overqualified employees usually possess more skills, knowledge, and abilities than the job requires, they may find it easy to deal with task problems and obstacles, and those past successful experiences can effectively improve employees’ confidence in their abilities needed to solve problems creatively. Second, since overqualified employees can perform their job better than others, they might receive trust, recognition, and praise from leaders or other coworkers (Maltarich et al., 2011), and this spiritual support will reduce their concerns about failure, and help to develop a positive evaluation of their CSE. Third, assessment of potential resource constraints also plays a critical role in formatting CSE. However, as employees who feel overqualified hold surplus skills, knowledge, and abilities, they believe that they have sufficient personal resources to perform creatively (Zhang et al., 2016). In summary, overqualified employees would be more confident to produce creative product/outcomes. Thus, this study proposes the following hypothesis:

**Hypothesis 1.** POQ is positively related to CSE.

### 2.2. POQ and deviant innovation behavior: The mediating role of CSE

As a result of heightened CSE, the current study further proposes that employees who feel overqualified may take more risky innovative actions to prove themselves. They may participate in deviant innovation behavior, which is defined as employees’ violation of managerial order or supervisory control to pursue new ideas (Mainemelis, 2010). Deviant innovation behavior is an extra-role behavior that can involve uncertainty and risk such that employees need to have a strong willingness to change their employment and be confident in their knowledge and skills to make such a change. Based on social cognitive theory (Bandura, 1986), this study proposes that POQ is likely to prompt deviant innovation behavior through CSE for the following reasons.

First, behavioral motivation of individuals comes not from the goals themselves, but from the self-evaluation that is made conditional on their fulfillment (Bandura, 1986, 1997). For example, when employees decide whether a new idea is valuable and worth pursuing, heightened CSE provides cognitive support and confidence, and thus they feel confident in their knowledge and abilities to generate valuable ideas that deserve pursuit. Moreover, once employees who perceive CSE commit themselves to valuable innovation, they seek self-satisfaction from achieving such innovations (Bandura, 1997). Therefore, overqualified employees with heightened CSE are more confident in implementing deviant innovation behavior.

Second, employees need permission to transform their new ideas into actions that generally require more cognitive resources, such as job-related knowledge, work time, and even limited resources within an organization (Mainemelis, 2010). However, employees who perceive themselves to be overqualified may feel that their organizations do not provide them with sufficient resources and opportunities for innovation (Luksyte et al., 2011), and thus they will have to utilize their own resources and implement the innovation secretly. According to social cognitive theory, employees with higher CSE are more likely to perceive challenges as opportunities and enhance their coping efforts when facing obstacles (Tierney and Farmer, 2002). Therefore, although employees engage in deviant innovation behavior that may be uncertain and risky (Mainemelis, 2010), higher CSE could better equip them to address the challenges and uncertainty faced when implementing new ideas in their workplace (Newman et al., 2018). Hence, this study proposes the following hypothesis:

**Hypothesis 2.** CSE mediates the relationship between POQ and deviant innovation behavior.

### 2.3. The moderating role of POS

Perceived organizational support refers to the extent to which employees develop general beliefs about how the organization values their contributions and cares about their wellbeing (Eisenberger et al., 1986). A recent review regarding POS suggested that organizational support not only implies a high-quality social exchange relationship between employee and organization, but also fulfills socioemotional needs of employees, increases their anticipation of receiving help when needed, and strengthens their reward expectations and self-efficacy (Kurtessis et al., 2017). In addition, according to social cognitive theory (Bandura, 1986, 1997), formation of CSE by an individual is also influenced by his/her estimation of resources that are available for generating and pursuing creative ideas and the actual acquisition of diverse information. Thus, this study supposes that the relationship between POQ and CSE will be moderated by the level of employees’ POS.

In settings where POS is high, organizations could provide more resource and support, overqualified employees usually have more trust in the organization, and know that their organization will help when they experience difficulties (Eisenberger et al., 1986). Thus, those support from the organization help to reduce obstacles during innovative process, then overqualified employees are less likely to have concern about the risks of pursuing innovative ideas, and will have more belief about their competence to achieve creative outcomes. Moreover, support and recognition from the organization could provide overqualified employees with positive feedbacks regarding their skills and abilities, which will further encourage them to consider their overqualification as effective capacities and potential worth, and will have more confidence in their role in bringing about valued innovation, so that they will develop more CSE (Bandura, 1997, 2001).

In contrast, in settings where POS is low, overqualified employees may feel angry about their employment situation. They may consider themselves to be less worthy organizational members, and that they do not make significant contributions to the organization, even though their qualifications exceed job requirements. Thus, the sense of not being supported by an organization could negatively impact the confidence of overqualified employees, to the extent that they would be hesitant to take risky moves. Based on the above analysis, this study proposes the following hypothesis:

**Hypothesis 3.** POS moderates the positive relationship between POQ and CSE, such that the positive relationship is stronger when POS is high.

Taken together, this study has proposed a mediating role for CSE in the relationship between POQ and deviant innovation behavior, and posit a moderating role for POS in the POQ-CSE link. These hypotheses suggest a moderated mediation model, that is, POQ has an indirect influence on deviant innovation behavior through CSE that is contingent upon POS. When overqualified employees perceive a higher level of POS, they are more likely to participate in deviant innovation behavior through enhanced CSE. On the other hand, when POS is low, the indirect influence of POQ on deviant innovation behavior through CSE would be weaker. Thus, this study proposes the following hypothesis:

**Hypothesis 4.** POS moderates the indirect effect of POQ on deviant innovation behavior *via* CSE, such that the indirect effect is stronger when POS is present.

**FIGURE 1:**
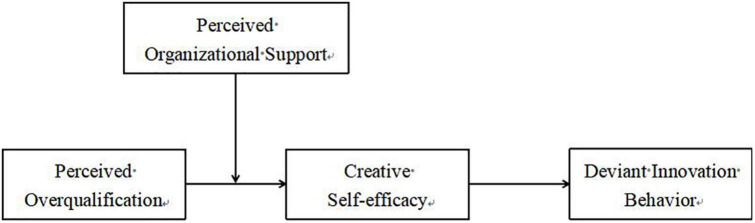
Theoretical model.

The conceptual model for this study is presented in Figure 1.

## 3. Materials and methods

### 3.1. Samples and data collection

To test our hypotheses, this study conducted questionnaire surveys and collected data from employees working at eight firms registered in service, manufacturing, and training industries that are located in Hebei and Guizhou provinces of China. Questionnaires were sent at two different time points to rule out the possible influence of common method variance. At time 1, employees were invited to report their POQ and demographic information. At time 2, which was 2 months later, employees were asked to rate their CSE, POS, and deviant innovation behavior. After excluding incomplete and invalid questionnaires, the study considered 286 employees. Of these, 58.4% were female, 33.9% were between 26 and 35 years old, and 46.9% had a bachelor’s degree.

### 3.2. Measures

As our research was conducted in China, this study followed the translation and back-translation procedure (Brislin, 1986) to translate all English measures into Chinese. All measures were established scales and were anchored on a five-point Likert scale ranging from 1 (strongly disagree) to 5 (strongly agree).

#### 3.2.1. POQ

This study adopted a 4-item scale developed by Johnson and Johnson (1996) to measure the employees’ POQ. A sample item was “I have more capacities than I need to do my job requirement.” In this study, the Cronbach’s alpha for this scale was 0.848.

#### 3.2.2. CSE

This study used a 4-item scale from Tierney and Farmer (2011) to measure CSE. A sample item was “I think that I can solve problem through some new ways.” In this study, the Cronbach’s alpha for this scale was 0.879.

#### 3.2.3. POS

Perceived organizational support was measured with Shanock and Eisenberger’s (2006) 6-item scale. A sample item was “My organization cares for my goals and I have a sense of value.” In this study, the Cronbach’s alpha for this scale was 0.914.

#### 3.2.4. Deviant innovation behavior

Deviant innovation behavior was evaluated with a 5-item scale developed by Criscuolo et al. (2014). A sample item was “I enjoy tinkering around with ideas that are outside the main projects I work on.” In this study, the Cronbach’s alpha for this scale was 0.924.

#### 3.2.5. Control variables

Similar to previous research (Schreurs et al., 2021; Zhang M. et al., 2021), this study also controlled for the gender, age and education of employees in this study.

## 4. Results

### 4.1. Confirmatory factor analysis

This study used AMOS 26.0 to conduct a series of confirmatory factor analyses (CFAs) to examine the discriminant validity of our four main variables, including POQ, CSE, POS, and deviant innovation behavior. Results showed in Table 1 revealed that a four-factor model fit the data best among all models tested (χ2 = 371.546, df = 146, χ2/df = 2.545, CFI = 0.939, TLI = 0.929, RMSEA = 0.074), which demonstrated empirical distinctions among the variables of this study.

**TABLE 1:** Results of confirmatory factor analysis.

Model	χ 2	Df	χ 2/df	CFI	TLI	RMSEA
Zero-factor model	3,874.538	171	22.658			
Four-factor model	371.546	146	2.545	0.939	0.929	0.074
Three-factor model	1,057.869	149	7.1	0.755	0.718	0.146
Two-factor model	1,744.745	151	11.555	0.57	0.513	0.192
Single-factor model	2,174.181	152	14.304	0.454	0.386	0.216

N = 286.

This study also calculated the heterotrait-monotrait ratio of correlations (HTMT) criterion (Henseler et al., 2015) to further examine the discriminant validity of our variables. Results shown in Table 2 suggest that the values of all the constructs were under the threshold point of 0.85, revealing satisfactory discriminant validity.

**TABLE 2:** Discriminant validity heterotrait-monotrait ratio of correlations (HTMT).

	POQ	CSE	POS	Deviant innovation behavior
POQ				
CSE	0.456			
POS	0.011	0.014		
Deviant innovation behavior	0.619	0.509	0.140	

N = 286.

### 4.2. Descriptive statistics

This study used SPSS 26.0 to generate the mean and standard deviation of each variable, and Pearson correlation analysis was used to explore the relationships between the variables. Table 3 shows the descriptive statistics including means, standard deviations, and correlations of all variables in this study. As shown in Table 3, POQ was positively correlated with CSE (*r* = 0.394, *p* < 0.01), and with deviant innovation behavior (*r* = 0.547, *p* < 0.01). Moreover, CSE was positively correlated with deviant innovation behavior at a significant level (*r* = 0.459, *p* < 0.01).

**TABLE 3:** Means, standard deviations, and correlations of all variables in the study.

Variable	*M*	SD	1	2	3	4	5	6	7
1. Gender	0.584	0.494	1						
2. Age	2.406	1.028	−0.019	1					
3. Education	2.724	0.873	−0.072	−0.102	1				
4. POQ	3.979	0.861	−0.035	−0.036	−0.051	1			
5. CSE	3.823	0.647	0.045	0.072	0.026	0.394**	1		
6. POS	4.158	0.686	0.063	0.118*	−0.123*	0.011	0.016	1	
7. Deviant innovation behavior	3.824	0.896	−0.087	0.075	−0.046	0.547**	0.459**	0.130*	1

N = 286. *p < 0.05; **p < 0.01. For “Gender,” 0 = male; 1 = female; For “Age,” 1 = ≤ 25 years-old, 2 = 26–35 years-old, 3 = 36–45 years-old, 4 = ≥ 46 years-old; For “Education,” 1 = High school, 2 = Specialty, 3 = Bachelor degree, 4 = Master’s degree.

### 4.3. Hypotheses testing

SPSS 26.0 was used to conduct hierarchical multiple regression analyses to test our hypotheses. Results are shown in Table 4. For Hypothesis 1, this study proposed a positive relationship between POQ and CSE. Results show that POQ was positively and significantly related to CSE (Model 2, β = 0.402, *p* < 0.001), thus supporting Hypothesis 1.

**TABLE 4:** Results of hierarchical multiple regression analysis.

	CSE				Deviant innovation behavior			
	**Model 1**	**Model 2**	**Model 3**	**Model 4**	**Model 5**	**Model 6**	**Model 7**	**Model 8**
Gender	0.05	0.066	0.066	0.065	−0.089	−0.067	−0.112*	−0.086
Age	0.076	0.094	0.093	0.09	0.068	0.092	0.033	0.065
Education	0.038	0.061	0.061	0.065	−0.046	−0.014	−0.063	−0.032
POQ		0.402***	0.402***	0.388***		0.547***		0.430***
CSE							0.464***	0.290***
POS			0.004	0.015				
POQ × POS		0.169**						
*R* ^2^	0.009	0.17	0.17	0.198	0.015	0.312	0.228	0.382
Δ *R*^2^	—	0.161	0.161	0.189	—	0.297	0.213	0.367
*F*	0.828	14.356***	11.445***	11.468***	1.429	31.928***	20.740***	34.647***

N = 286. *p < 0.05, **p < 0.01, ***p < 0.001.

Hypothesis 2 predicts that CSE mediates the relationship between POQ and deviant innovation behavior. Both POQ (Model 6, β = 0.547, *p* < 0.001) and CSE were positively associated with deviant innovation behavior (Model 7, β = 0.464, *p* < 0.001; Table 4). However, after adding CSE, the positive influence of POQ on deviant innovation behavior decreased (Model 8, β = 0.430, *p* < 0.001). To further examine the mediating effect of CSE, this study conducted a bootstrapping analysis, and found a significant indirect effect of POQ on deviant innovation behavior *via* CSE (indirect effect = 0.121, 95% CI = [0.080, 0.175]). Thus, Hypothesis 2 was supported.

**FIGURE 2:**
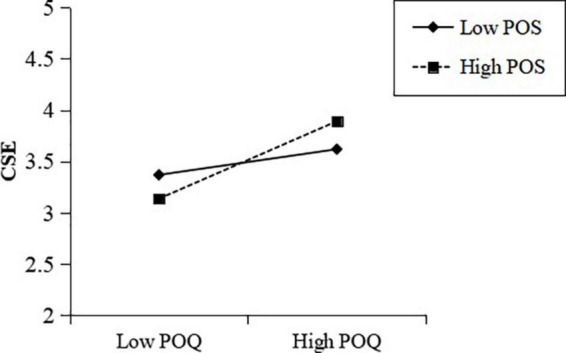
Moderating effect of POS on the relationship between POQ and CSE.

As shown in Table 4, the interaction of POQ and POS was positively related to CSE (Model 4, β = 0.169, *p* < 0.01), suggesting the moderating effect of POS on the relationship between POQ and CSE. This study then applied PROCESS 3.5 macro for SPSS to examine the moderating role of POS. Results shown in Table 5 suggested that, when POS was high, the effect of POQ on CSE was 0.377 (95% CI = [0.266, 0.492]), when POS was low, the effect of POQ on CSE was 0.125 (95% CI = [0.029, 0.221]), and there was a significant difference for this effect when comparing conditions of high and low POS (difference = 0.252, 95% CI = [0.094, 0.382]). In a simple slope analysis (Figure 2), when POS was high, the effect of POQ on CSE was stronger (*b* = 0.377, *t* = 7.179, *p* < 0.001), compared to when POS was low (*b* = 0.125, *t* = 2.195, *p* < 0.05), thus providing support for Hypothesis 3.

**TABLE 5:** Results of the conditional indirect effects.

Variables	POQ → CSE	Direct effect	Indirect effect	Total effect
High POS	0.377*	0.170*	0.173*	0.342*
	[0.266, 0.492]	[0.025, 0.351]	[0.118, 0.247]	[0.189, 0.522]
Low POS	0.125*	0.600*	0.057*	0.657*
	[0.029, 0.221]	[0.442, 0.735]	[0.016, 0.102]	[0.494, 0.800]
Differences	0.252*	−0.431*	0.116*	−0.315*
	[0.094, 0.382]	[−0.628, −0.208]	[0.045, 0.186]	[−0.532, −0.087]

N = 286. 95% confidence intervals were reported in the parentheses. *p < 0.05.

This study also tested conditional indirect effects (Hypothesis 4) using the PROCESS 3.5 macro for SPSS. The indirect effect of POQ on deviant innovation behavior *via* CSE was significant (indirect effect = 0.173, 95% CI = [0.118, 0.247]) when high POS was present, and this indirect effect was also significant (indirect effect = 0.057, 95% CI = [0.016, 0.102]) when POS was low (Table 5). However, there was a significant difference for the indirect effect when comparing conditions of high and low POS (difference = 0.116, 95% CI = [0.045, 0.186]). Thus, Hypothesis 4 was supported.

## 5. Discussion

The aim of the present study was to explore the underlying mechanisms by which employees’ POQ influences deviant innovation behavior. The current study demonstrated that POQ could strengthen an employee’s CSE, and positively influence deviant innovation behavior through the mediating role of CSE. Moreover, POS moderates the relationship between POQ and CSE, as well as the indirect relationship between POQ and deviant innovation behavior *via* CSE. When POS is high, this indirect effect is stronger than when POS is low.

### 5.1. Theoretical implications

Results of the present study make several contributions to existing research on POQ. First, this study advances our understanding of the consequences of POQ. Early research mainly focused on negative outcomes of POQ (Luksyte et al., 2011; Liu et al., 2015), and generally assumed that overqualification fosters undesirable work conditions. In fact, POQ may provide a major impetus for employees to participate in proactive or innovative behaviors (Zhang et al., 2016). Our results make a further improvement on this conclusion by linking POQ with deviant innovation behavior, a form of proactive behavior that can benefit employees’ organizations and transform their surplus qualifications into competitive advantages. Our results suggest that overqualified employees may proactively seek new professional development opportunities to prove their abilities.

Second, this study provides a new insight for understanding the mechanism of how POQ can influence employee behaviors by identifying CSE as a mediator. Prior research mainly draws from relative deprivation theory to suggest that overqualification could invoke employees’ feeling of relative deprivation, and will result in poor job attitudes, more turnover intention, and higher levels of counterproductive behaviors (Harari et al., 2017). Although the relative deprivation perspective did help to understand the influences of POQ, it only reveals the negative aspect of POQ. Based on social cognitive theory, this study suggests that POQ will also enhance employees’ positive self-evaluation about their creative ability, thus encouraging them to actively participate in innovative activities even though they may cross some organizational lines. Thus, our research advances our understanding of the causal process of POQ on employee behaviors, and offers a new perspective to explore other potential outcomes of POQ.

Third, by incorporating POS as a moderator of the effect of POQ on deviant innovation behavior through CSE, our results reveal under what circumstances the effects of POQ on deviant innovation behavior would be strengthened. Specifically, our findings demonstrate that POS can serve as a boundary condition that amplifies the positive influence of POQ. In other words, when overqualified employees feel that they are highly valued and recognized by organizations, their belief in their abilities to make a change would be further activated, and they would be more likely to neglect potential risks to conduct deviant innovation behavior.

### 5.2. Practical implications

First, the current study informs organizational managers and leaders about which employees are likely to conduct deviant innovation behavior in organizations. Although deviant innovation behavior could bring benefits to an organization in the form of new breakthroughs, such behavior is also uncertain and risky in nature and could consume resources. Thus, if organizations lack sufficient resources to innovate, managers and leaders should pay more attention to employees who feel themselves to be overqualified. HR departments should carefully select fit-match employees during the recruitment process, and relocate overqualified employees to suitable positions to reduce their POQ. Second, our results suggest that employees’ POQ could increase their CSE, which usually promotes innovative behavior. Thus, managers and leaders should accurately and specifically articulate and communicate their innovation expectations to overqualified employees, so that those overqualified employees who want to prove their abilities and values would have a more clear and appropriate direction to innovate. Finally, although our study was conducted in China, which is a developing country, as the continued downturn of the global economy and the fierce competition in the labor market, underemployment has become a global employment phenomenon (Chu et al., 2021), and could be more widespread in developed countries (Erdogan et al., 2011). Thus, our research may also provide managers in developed countries a new solution to stimulate the potential beneficial effects of overqualification.

### 5.3. Limitations and future research directions

Our study does have some potential limitations that should be addressed in future research. First, the data collected for this study were self-reported and may have some common method biases. Although this study collected data at two different time points to reduce these potential influences, respondents may be affected by social desirability bias. To address this issue, future research could use alternative methods, such as collection of multi-source data. Second, our research was conducted in China, which has a higher level of power distance and collective orientation such that Chinese employees’ perceptions of overqualification may differ from that for employees in Western countries. Future research could be conducted in countries having different cultural backgrounds to examine the generality of our findings. Third, although our research examined the indirect effect of POQ on deviant innovation behavior through CSE, and POS as a boundary condition. However, other factors may affect this indirect relationship as well. Thus, future research could explore how other potential moderators, such as personality traits and leadership styles, would influence this relationship.

## 6. Conclusion

This study used a questionnaire survey method to explore the influence of employee POQ on deviant innovation behavior, as well as its mechanism and boundary conditions. Results of this study showed that POQ can indirectly impact deviant innovation behavior through CSE. This study also showed that when the level of employee POS is higher, the effect of POQ on CSE is stronger, and the indirect effect of POQ on deviant innovation behavior *via* CSE is also stronger. These results extend our understanding of the consequences of POQ from a social cognitive theory perspective. Taken together, organizational managers and leaders should pay more attention to overqualified employees, and take proper actions to guide them to achieve innovative results needed by organizations.

## Data availability statement

The raw data supporting the conclusions of this article will be made available by the authors, without undue reservation.

## Ethics statement

Ethical review and approval was not required for the study on human participants in accordance with the local legislation and institutional requirements. Written informed consent from the [patients/participants OR patients/participants legal guardian/next of kin] was not required to participate in this study in accordance with the national legislation and the institutional requirements.

## Author contributions

LL and YZ designed and adopted the study and wrote the manuscript. LL and TL wrote the manuscript. All authors approved the submitted version.
